# Preparation and characterization of coffee hull fiber for reinforcing application in thermoplastic composites

**DOI:** 10.1080/21655979.2019.1661694

**Published:** 2019-09-17

**Authors:** Zhihao Wang, Lemma Dadi Bekele, Yue Qiu, Yifan Dai, Shiqiang Zhu, Surendra Sarsaiya, Jishuang Chen

**Affiliations:** a College of Biotechnology and Pharmaceutical Engineering, Nanjing Tech University, Nanjing, Jiangsu, China; b Bioresource Institute for Healthy Utilization (BIHU), Zunyi Medical University, Zunyi, Guizhou, China

**Keywords:** Coffee hull fiber, HDPE, Thermoplastic Composite, mechanical properties, fiber treatment

## Abstract

Nowadays, there is an increasing concern toward substituting the scarce wood fibers with alternative lignocellulosic fibers that originate from crop residue to reinforce biocomposites. In this paper, the potential application of coffee hull (CH) of the reinforced polyethylene (PE) matrix composites was studied for the first time. Experiments of composite that enhanced with CH on mechanical properties, hydroscopicity, thermogravimetric analysis, fiber treatment, and microstructures were tested in this study. The PE matrix was reinforced with varying volume fractions of CH and was studied. The results show that incorporation of coffee hull markedly improved the mechanical properties of the reinforced high-density polyethylene (HDPE) matrix composites. Micrographs show a strong interfacial adhesion between the CH fiber particles. This property may be the main reason for the stability between composites. At the same time this work investigated the effect of different treatments on the mechanical properties and water absorption behavior of composites. The fiber surface treatments were done using active chemicals such as calcium hydroxide (Ca(OH)_2_), silane coupling agent (SCA), maleic anhydride grafted polypropylene (MA-g-PP), stearic acid (SA), ethylene bis stearamide (EBS) and the combination (MA-g-PP, SA, EBS). The results show that (Ca(OH)_2_)treatment is the best way to improve its properties. Probably because attributed to removal of surface active functional groups (-OH) from the CH fiber and induction of hydrophobicity that in turn improved the compatibility with the polymer matrix. As a result, the use of coffee hull in composites could have great significance for the industry.

## GraphicalAbstract

**Figure:**
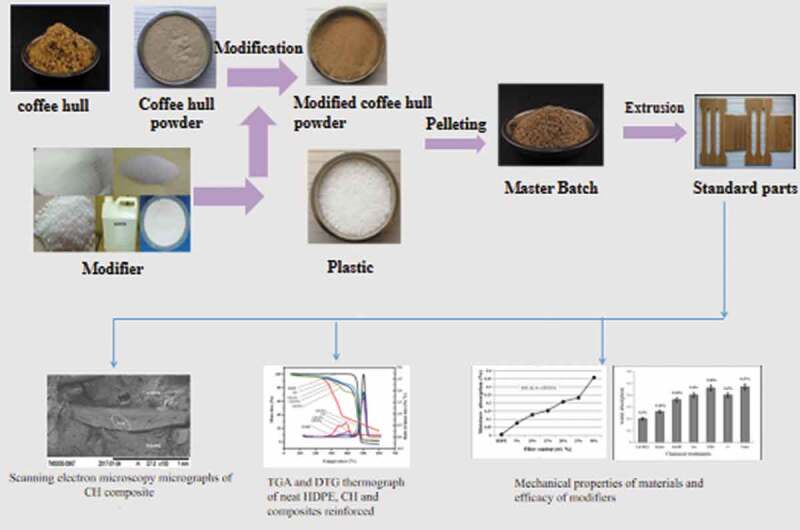

## 1. Introduction

Recent days, concerns to protect the environment and interests to save the depleting nonrenewable fossil-based resources have created the impetus to both researchers and industries to seek for alternative ways to use environmentally friendly, nonabrasive, nontoxic and renewable natural fibers as substitute reinforcing phase in composite materials. During the past several decades a number of far-reaching studies on the potential use of many different natural fibers in polymer matrices composites have been under taken and promising findings were reported. The mechanical, thermal and weathering properties of wood fiber reinforced polymer matrices with or without fiber treatments have been widely described [1,2]. Several other research work has also been conducted on potential use of non-wood natural fiber types in polymer composites. Reports on applications of various kinds of baste fibers including sisal [3,4], hemp fiber [5], palm fiber [6,7], rape fiber [8], and flax fiber [9] in composite materials synthesis showed the appreciable quality of the respective fibers as a reinforcing phase in composites. In addition, banana [10,11] sugarcane bagasse [12,13], rattan fiber [14], coir [15,16], jute fiber [17], snake grass [18], doum fiber [19], henequen fibers [20],and wheat straw [21] are annual plant biofibers widely investigated for their potential use as a reinforcing phase in polymer matrix composites. The majority of these investigations demonstrated the potential applicability of the respective fibers in the synthesis of composites and the worth of these natural fibers against their synthetic counterparts in reinforced polymer composites. Currently, there is increasing use of agricultural biomasses such as straw and husks for the production of composite materials [22]. But there have been few reports of have good lignocellulosic properties coffee hull fibers.

Coffee is one of the most consumed beverages in the world and the second most globally traded commodity next to petroleum earns huge foreign currency to coffee producing countries including Brazil, Vietnam, Indonesia, Colombia, Ethiopia. But the coffee bean production process generates large amounts of residue. Almost all of which is disposed of through burning or dumping as landfill or disposal directly in to water bodies. Coffee hull is described as a fiber rich agricultural residue consisting of various proportions of neutral and acid detergent fibers in amounts of 88% and 67%, respectively [23]. Cerda A, and Gea T [24]in their work indicated that coffee hull fiber role of microbial diversity. Coffee hull from Arabica coffee type is constituted of 41–43%, 5–10% and 1–3% of cellulose, hemicellulose and lignin components, respectively [25]. In other reports, it was shown that coffee hull constitutes of lignin and ash amounted to 18% and 5%, respectively [26]. As a cellulose-rich material, coffee hull can be considered a potential source of reinforcing phase in thermoplastic matrix composites. The higher levels of lignocellulosic components and cellulose in particular, make coffee hull an attractive ideal candidate fiber in the fabrication of composites by blending it with polymer matrices. In favor of its structure and high cellulose content, coffee hulls has been considered in synthesis of particleboard. It has been observed that coffee hull can replace as high as 50% of wood fiber in the production of resin bounded natural fiber-based particleboards [25]. Apart from serving as a substitute for conventional fibers, the use of natural fibers in the composites areas has paramount importance in mitigating the environmental pollution arising from the disposal by incineration and land filling of agroindustry and agroforestry lignocellulosic biomass.

Moreover, in the preparation process of biocomposites, CH fibers tend to aggregate with each other, forming fiber clusters, causing stress concentration and increasing the probability of defects and resulting in the decline of mechanical properties of the biocomposites. Therefore, to improve the interface compatibility and mixing uniformity are the key to producing excellent bio-composites [27]. The main objective of this research work was to investigate the potential use of coffee hull as reinforcement phase in HDPE composite. Stem CH (coffee hull)/HDPE (high density polyethylene) composites are prepared and studied. The mechanical properties of the resulting composites, the control and processing of CH are examined from a feasibility, technical and theoretical basis. At the same time, the surface modification method was used to solve the defects of the composite, and the best formula was found.

## 2. Materials and methods

### 2.1. Materials

The CH used in this work was obtained from the Arabica coffee processing union in Mizan-Aman town, southwest Ethiopia. As the matrix material, HDPE (5000s, melt flow index of 0.90 g/min, density 0.95 g/cm3, melt point 130°C) was manufactured by Sinopec Yangzi Petrochemical Co., Ltd. China. As chemical modifiers, Ca(OH)_2_ (Jining Yinhe environmental protection material Co., Ltd.), SCA (KH-550, Nanjing Chuangshi Chemical Co., Ltd.), MA-g-PP (Shenzhen Haian Plastic Chemical Co., Ltd.), SA (Shanghai beite chemical Co., Ltd.) and EBS (Changzhou Kesai Success Plastics Materials Co., Ltd.) were to improve the interface compatibility between materials.

### 2.2. Fiber preparation

The CH used for this study was washed with distilled water to remove surface dust and dried them with drying oven (GZX-9246,Shanghai Boxun Medical biological instrument Co., Ltd. China) under 80°C to assure its moisture content below 3 wt. %. The dried CH was pounded by using a rotary hammer mill (TQ-1000Y, Yongkang tianqi shengshi industry and trade Co., Ltd, China) and sieved with a sieving machine (AS 300 Control, Retsch, Germany). The CH powder with size 20 to 60 meshes was used for this study.

### 2.3. Chemical modification of the fiber

The pounded CH was stirred with 3 wt. % SCA by using a high-speed mixer (SHR-50A, Zhangjiagang Gelan Machinery Manufacturing Co., Ltd. China) and the parameter setting as follows: the temperature is 80°C while the revolving speed is at 1500 rpm/min for 20 min. The modified CH powder was dried to the moisture content of around 3 wt. % prior to further processing.

The clean dried CH powder was immersed in 3 wt. % Ca(OH)_2_ for 6 h. After immersion, the soaked CH were thoroughly washed with distilled water till the pH of the water came from fiber became neutral. The pH of the water was checked with litmus paper. The alkaline treated and washed CH was finally dried at 80°C and pounded in size between 20 and 60 mesh by using the aforementioned sieving machine.

As additional modifiers in this study, MA-g-PP, SA, EBS or their combination that by adding them directly was to modify the CH fiber. Then, the mixture was mixed with pure HDPE by double roll open rubber mixer (XK160, Yangzhou jiangdu ruijin test machinery Co., Ltd. China). Table 1 shows that specimen types with different chemical treatment.

**Table 1.** Chemical treatments of CH.

Specimens	Ca(OH)_2_(%)	SCA(%)	MA-g-PP(%)	SA(%)	EBS(%)
S1	3	-	-	-	-
S2	-	3	-	-	-
S3	-	-	3	-	-
S4	-	-	-	3	-
S5	-	-	-	-	3
S6	-	-	1	1	1
S7	-	-	-	-	-

### 2.4. Preparation of composite

#### 2.4.1. Preparation of composite with different volume fractions

Pounded CH powder at varying content (5 10, 15, 20, 25, 30, 35 and 40 wt. %) was thoroughly compounded with the HDPE matrix and 3 wt. % MA-g-PP aimed to obtain the effects of CH content to its properties. The melt-mixing method was employed to homogeneously blend the components by using double roll open rubber mixer which operated at a temperature of 165°C and for a period of 7 min. The blended homogeneous mixture was then put in stainless squared panels and compression casted into 200 mm × 200 mm × 4 mm by press vulcanizer (RJ-25T, Jiangsu Ruijin Test Machinery Co., Ltd. China). The setting parameter of this machine as follow: the temperature is 190°C and the pressure is 15 MPa. Compression was repeated by inverting the panels at 5 min intervals to assure complete amalgamation and this step continues 3 times for each composite.

#### 2.4.2. Preparation of composite with different modifier treatmen

In case of chemical modification by MAPP, stearic acid, EBS or the combination of the three chemicals, composite preparation involved direct blending either of the chemicals, with the CH flour and polyethylene matrix. Accordingly, either of the chemicals, CH flour and HDPE matrix were mixed at 3:20:67 ratio, and homogeneously melt-mix blended using two counter rotating twine cylindrical hot-press mixers, each of which operated at a temperature of 165°C, for a period of 7–10 min. Silane treated CH flour and HDPE matrix were mixed at 23:76 ratios and homogeneously compounded using the aforementioned machine and time. Alkaline treated CH flour and HDPE were mixed with 20:80 ratios prior to blending. The homogeneously blended composite was then compression casted into squared panels using an SLB-25-D350 Carver hydraulic high pressure compression hot press machine operated at 190°C and 15 MPa compression temperature and pressure, respectively.

### 2.5. CH fiber composites characterization

#### 2.5.1. Water absorption test

The water absorption test was carried out according to ASTM 570-D98: standard test methods for water absorption of plastics. The samples were dried at 105°C for 24 h, then the prepared samples were weighed with a 0.001 g precision balance (BSA224S, Beijing Sartorius Scientific Instruments Co., Ltd. China) and immersed in distilled water at 23°C for 24 h. At end of immersion period, removed the samples from the beaker and wiped off the surface water with a clean and dry towel prior before weighing them again. Percentage increase in weight during immersion, calculated to the nearest 0.01% as follows:

$$W,\%=\left(\frac{W_{2}-W_{1}}{W_{1}}\right)\times 100\%$$

where W is water absorption. W1 is the conditioned weight of the composite. W2 is the wet weight of the composite.

#### 2.5.2. Thermogravimetric analysis (TGA)

Thermal stability of the HDPE&CH composites with 10, 20 and 30 wt. % CH fiber were evaluated by using a thermogravimetric analyzer (Netzsch STA 449F3, Düsseldorf, Germany). The tests were performed in a nitrogen atmosphere under a flow rate of 60 ml/min, to prevent the occurrence of oxidation. Approximately 20 mg of each sample was placed on a platinum pan and heated from a temperature of 30°C to 600°C at a heating rate of 10°C/min to yield a maximum temperature decomposition peak.

#### 2.5.3. Scanning electron microscopy (SEM)

SEM of 20 wt. % CH reinforced composites was used to evaluate the fiber to polymer matrix interfacial bonding strength. A scanning electron microscope (TM3000, Hitachi, Japan) was used in this investigation. The sample which has been ruptured by liquid nitrogen were mounted on conductive adhesive tape and the fracture surfaces were sputter coated with gold prior to microscopic examination.

#### 2.5.4. Tensile testing

The tensile properties of the composites were determined by a universal testing machine (UTM-1422, Chengde Jinjian Testing Instrument Co., Ltd. China) and follow the standard with ASTM d638-10. The samples were cut into 150 × 50 × 4 mm conditioned composite plates and which appearance like ‘dog-bone’ was made by dumbbell specimen machine (XYZ-20, Chengde Jinjian Testing Instrument Co., Ltd. China). Each treatment was represented by five identical ‘dog-bone’ specimens to determine their tensile properties.

#### 2.5.5. Flexural testing

According to ASTM D790-07 procedures, through the three point bending method for determining the bending properties of composites [28].Using the above machine test average of 1.9 mm/min speed and measuring element of 10 kn load test conditions for each composite plate cutting into size (80 mm × 10 mm × 4 mm) strip using profile sample maker (XXZ-II, Chengde Jinjian Testing Instrument Co., Ltd. China). Each composite board with five short rod samples cutting to represent their respective processing method.

## 3. Result and discussion

### 3.1. Moisture absorption

**Figure 1.**
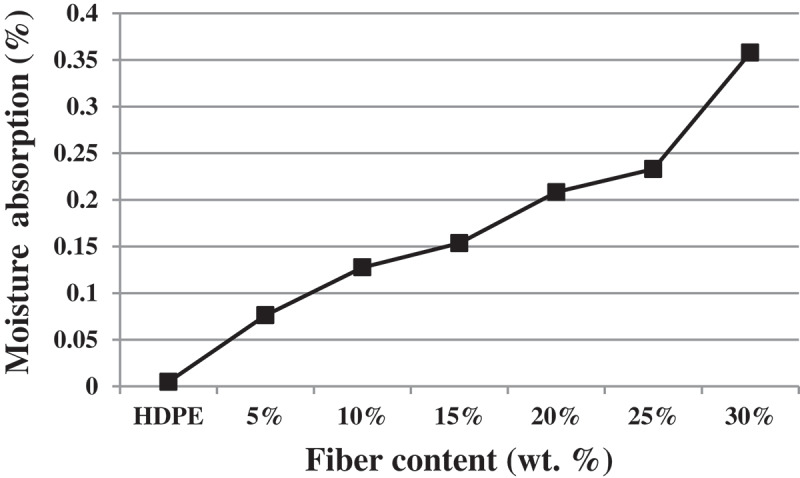
Water absorption values by neat HDPE, and composites reinforced with varying amount of CH after 24 h of water immersion.

The water absorption values of the CH/HDPE composites reinforced with varying fiber content are presented in Figure 1. An increase in fiber volume fraction of CH slightly increased the tendency of the corresponding biocomposite to absorb more water.

Moisture absorption increased from 0.076wt. % value at 5wt. % fiber content to 0.4wt. % value at 30wt. % CH content. Dhakal and his coworkers [29] in their investigation on the impact of fiber loading on the water absorption of hemp fiber reinforced unsaturated polyester composites also showed that water absorption increased constantly with increase in fiber content. In another study, it has been described that a higher rate of moisture absorption recorded among hybrid composite containing high fiber content in roselle/sisal polyester-based hybrid composites and moisture environment was shown to decrease in tensile and ﬂexural strength [30]. Similar findings were reported by Mohamed et al. [31]. On moisture absorption of pineapple-leaf ﬁber reinforced low density polyethylene (LDPE) composites.

The apparent increase in the tendency of water uptake with increased fiber volume fraction can be explained in terms subsequent increase in the number of hydroxyl functional groups in the structure of the composite materials rendering them exhibit high hydrophilic behavior [32].

**Figure 2.**
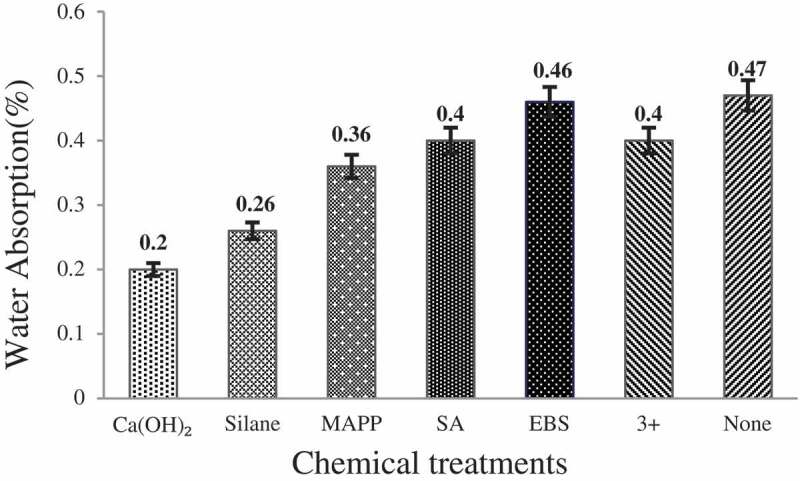
Moisture absorption rates of CH composites.

The effect of various chemical treatments on water resistance of the CH flour reinforced HDPE matrix composites are presented in Figure 2. The general data can be summarized as the resistance against water absorption developed among the composite when the fiber is modified by Ca(OH)_2_ > silane > MA-g-PP > combination > EBS > SA > untreated fiber composites.

The main reason is that silane is suitable for PE polycondensation grafting reaction with wood powder and may be added with HDPE, which has a strong coupling effect and makes the interface compatibility significant [33].

### 3.2. Thermogravimetric analysis (TGA)

**Figure 3.**
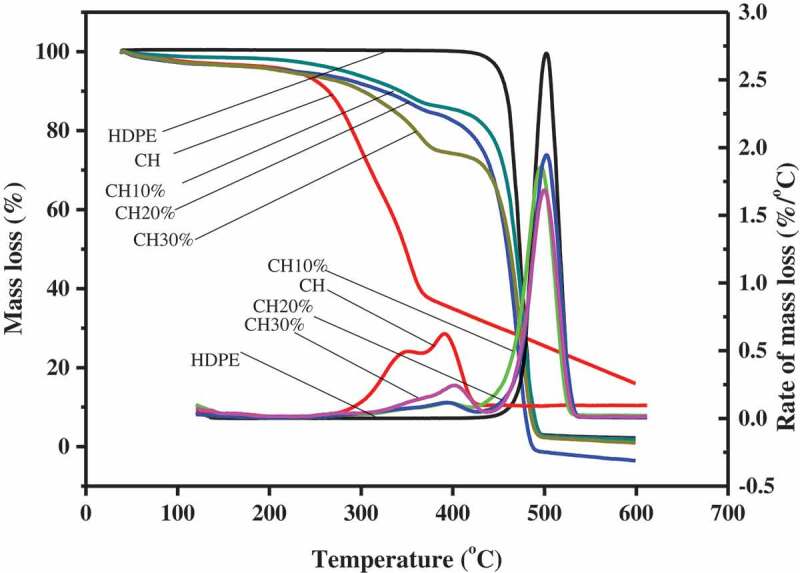
TGA and DTG thermograph of neat HDPE, CH and composites reinforced with 10wt. %, 20 wt. % and 30 wt. % CH.

Investigation of TGA was conducted to determine degradation temperatures of the CH, neat matrix and reinforced composites. Determining the thermal degradation properties of materials is necessary to gauge the materials ability to withstand the processing temperature. The TGA and DTG thermograph curve of neat HDPE, CH and composites reinforced with 10wt. %, 20wt. % and 30wt. % CH flour is shown in Figure 3. Mass loss from thermal changes in CH took place in the ranges of 38°C to 119°C, 119°C to 242°C and 242°C to 372°C. Thermal degradation of CH was started at 242°C and most of the mass lost at around 398°C. The highest rate of mass loss in the fiber was occurred at 347°C which is the temperature at which most of the lignocellulosic fiber components undergo degradation. The initial thermal degradation temperature of 242°C shows that CH can withstand the processing temperature of composites that is usually below 200°C. The thermal degradation of the CH/HDPE composite took place in two phases from 260°C–395°C to 397°C–470°C. The mass loss in the first temperature ranges can be due to the thermal degradation of lignocellulosic components of CH mainly cellulose and lignin, respectively [34]. Thermal degradation in the second thermal range can be attributed to mass loss from breaking down of HDPE and some lignin components of CH and it also corresponds to the thermal degradation value of neat HDPE [35]. The comparative analysis of the thermal degradation of composites and neat HDPE revealed that the incorporation of the fibers with the matrix decreased the degradation temperature of the composites compared to the neat polymer matrix. Variation in fiber content resulted in a remarkable difference in the thermal degradation rate of the corresponding composites, in that those with high fiber content exhibited substantially high degradation temperature ranges compared to the one reinforced with lower CH contents.

The DTG shows that CH and neat HDPE matrix each has one prominent peak at 347°C and 473°C, respectively. The DTG of neat HDPE peaked at 473°C that indicates the temperature at which maximum rate weight loss occurred in the matrix. Composites reinforced with 10wt. %, 20wt. % and 30wt. % CH flour demonstrated two peaks each at around 358°C and 473°C that are attributed to thermal decomposition of CH flour components and HDPE matrix, respectively. This showed that the incorporation CH flour into the matrix changed the thermal degradation pattern of the composite. Moreover, the higher initial thermal decomposition point of 249°C recorded for the CH fiber show that CH can tolerate the composite processing temperature used by the extrusion machine. Furthermore, the thermal degradation points gauged for each components of the composite demonstrated all the components can withstand the extrusion temperature used in composite preparation.

### 3.3. Scanning electron microscopy analysis

**Figure 4.**
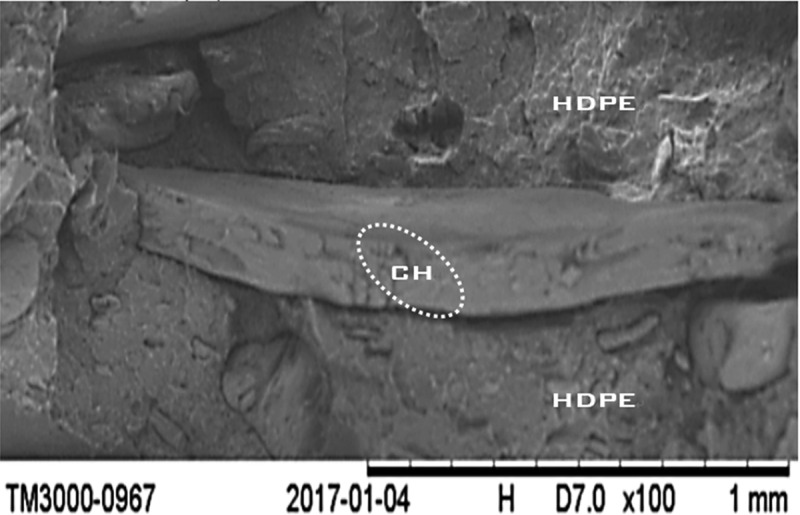
Scanning electron microscopy micrographs of CH composite.

Figure 4. shows the SEM micrographs of the cryo-fractured surface of 20wt. % CH reinforced HDPE matrix composites. Micrographs show a strong interfacial adhesion between the CH fiber particles and the polymer matrix characterized by the absence of fiber pullout and matrix tearing. This strong interaction can be associated with better mechanical interlocking of the two phases or with enhanced chemical bonding between fibers and the polymer matrix. Fibers in the matrix play an important role in stress energy transfer and enhance the mechanical properties of composite materials.

### 3.4. Tensile properties

**Figure 5.**
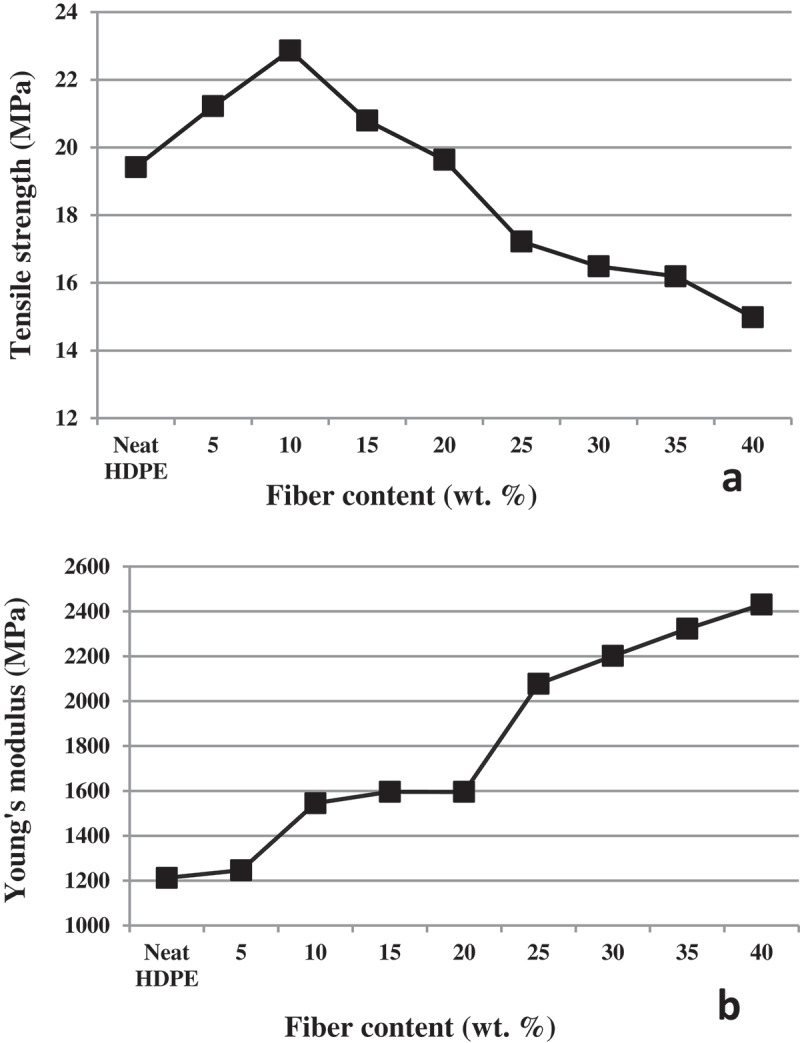
(a) Tensile strength of neat HDPE and composites reinforced with varying content of CH; (b) Young’s modulus of neat HDPE and composites reinforced with varying content of CH.

The tensile properties of the CH/HDPE composites at varying fiber content are shown in Figure 5(a,b). The tensile strength of the CH flour reinforced composite was found to increase from its value for neat polymer matrix to the10wt. % CH reinforced matrix composite and then started to decrease afterward as fiber volume fraction increases. Figure 5(a) shows the tensile strength value of the composites reinforced with varying content of CH. As compared to the tensile strength value of 19.42 MPa in neat HDPE, the tensile strength of the composites reinforced with 10 wt. % volume fraction of CH flour was found increased by 17.8 wt. % (22.86 MPa). The tensile strength values were found to gradually decrease as the fiber loading increased beyond the 10 wt. % CH content. The 22.86 MPa tensile strength value recorded for CH reinforced composite in this work was comparatively higher than the tensile strength value reported for polyethylene matrix composite reinforced with equal amount of rice husk [36]. The gradual decrease in tensile strength values beyond its peak at 10wt. % fiber content can be attributed to the development of weak interfacial interaction between the fiber and the matrix [37].

The Young’s modulus values of polyethylene matrix composite reinforced with different content of CH is shown in Figure 5(b). The Young’s modulus values of the composites were steadily increased with the increase in fiber volume fraction. A 100.4wt. % increase in Young’s modulus value from 1212 MPa for neat HDPE to 2430.4 MPa for composites reinforced with 40wt. % fiber volume fraction was recorded. The increase in Young’s modulus value with fiber volume fraction can be associated with the subsequent increase in stiffness of the reinforced polymer matrix composite [38].

**Figure 6.**
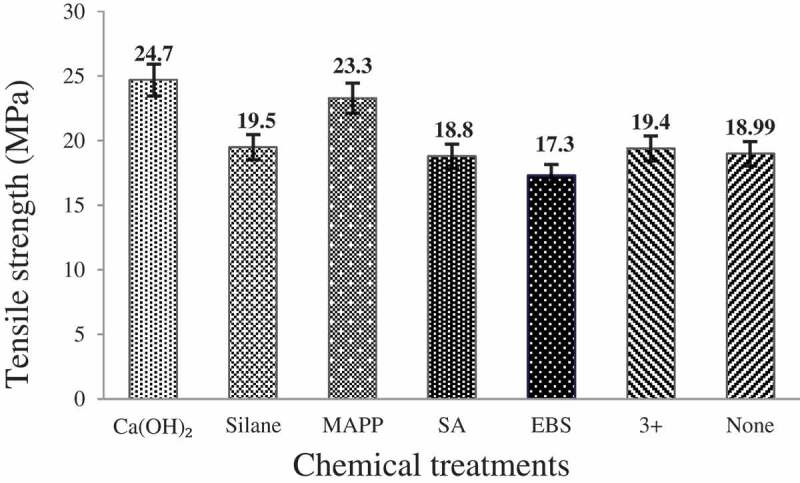
The effect of various chemical treatments on the tensile strength of CH reinforced composites.

The effect of chemical treatment on the tensile strength values of CH flour reinforced composite is presented in Figure 6. The general data can be summarized as Ca(OH)2 > MA-g-PP > silane > combination > untreated> SA > EBS. The improvement in tensile strength of the composite can be attributed to better fiber to matrix interfacial interaction and increased adhesion following the chemical treatments. The highest improvement in tensile strength among composites treated with Ca(OH)_2_ may be attributed to removal of some CH fiber components that otherwise their presence decrease the strength of interfacial interaction and adhesion between the fiber and matrix. Furthermore, an increase in hydrophobicity of the fiber can also be the meaningful explanation for the improved tensile strength value [39]. EBS based treatment of CH fiber drastically decreased the tensile strength of composites reinforced with the respective fiber with the value even lower than those of composites reinforced with untreated CH flour. It may be that the introduction of EBS has hardened the structure of some components of the fiber [40].

**Figure 7.**
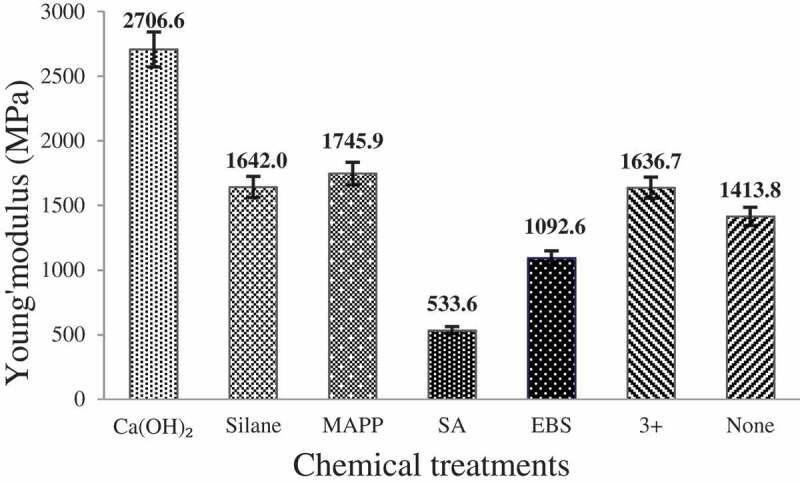
The effect of different chemical treatments on the Young’s modulus of CH reinforced composites.

The effect of various chemical treatments on the Young’s modulus of CH flour reinforced composites is shown in Figure 7. The general data can be summarized as: Ca(OH)_2_ > MA-g-PP > silane > combination > untreated> EBS > SA.

From the overall results of tensile strength and Young’s modulus value of the composites, it can be concluded that Ca(OH)_2_ treated composite demonstrated the highest performance in terms of tensile properties. The highest tensile strength and Young’s modulus values in Ca(OH)_2_ treatment can be attributed to removal of surface active functional groups (-OH) from the CH fiber and induction of hydrophobicity that in turn improved the compatibility with polymer matrix. In addition, Ca(OH)_2_ treatment induces fiber surface roughness through removal of amorphous components and supported mechanical interlocking between the fiber and the matrix.

### 3.5. Flexural property

**Figure 8.**
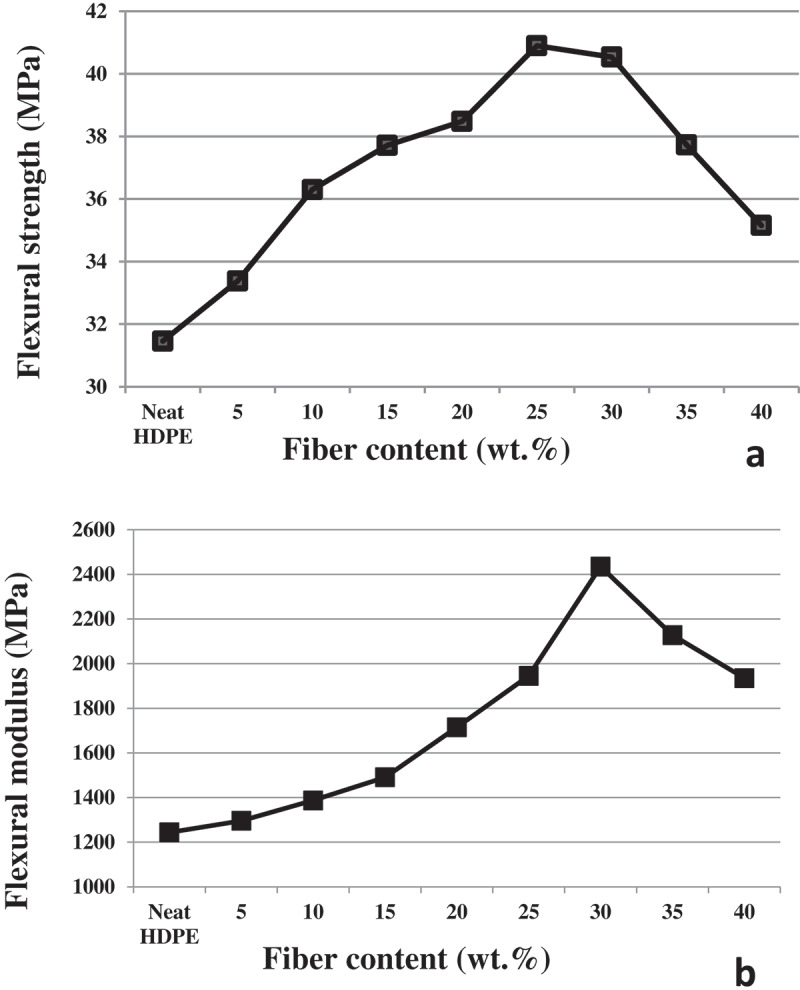
(a) Flexural strength of neat HDPE and composites reinforced with varying content CH (b) flexural modulus of neat HDPE and composites reinforced with varying content CH.

The flexural properties including flexural strength and flexural modulus of the HDPE/CH composites are shown in Figure 8(a,b). As it can be seen in Figure 8(a), the flexural strength values were progressively increased as the CH volume fraction increases peaking at 25 wt. % CH content and then decreased afterward. The reinforcement improved the flexural strength value by 29.8 wt. % from 31.5 MPa for neat HDPE to 40.9 MPa for composites reinforced with 25wt. % CH flour, respectively. The flexural moduli steadily increased with increment in the CH volume fraction in the composites Figure 8(b). The flexural modulus of the composite was increased from 1243 MPa for neat HDPE to 2435 MPa for composite reinforced with 30 wt. % CH contents, which accounts for a 95.8 wt. % increase.

The reason may be adding less fiber is not sufficient to form a continuous phase, with the fibers tending instead to be distributed as islands in the polymer matrix, with uneven distribution and large distances between the islands [41]. Due to the inability to form rigid particles restraining each other, the resulting composites are easy to fracture, and their corresponding mechanical properties decrease under the action of an external force [42]. Increasing lignocellulose loading gradually, because of the low density and small volume, narrows the distance between the lignocellulose fibers and improves the fiber and plastic distribution gap and induces the fibers to contact with each other, interacting by crossing or even winding, which will improve the properties of the composites [43]. However, the close contact between the fibers gives the composites a strong resistance, and the need to overcome the strong friction between the fibers means that the corresponding bending and tensile properties are relatively large [44].

**Figure 9.**
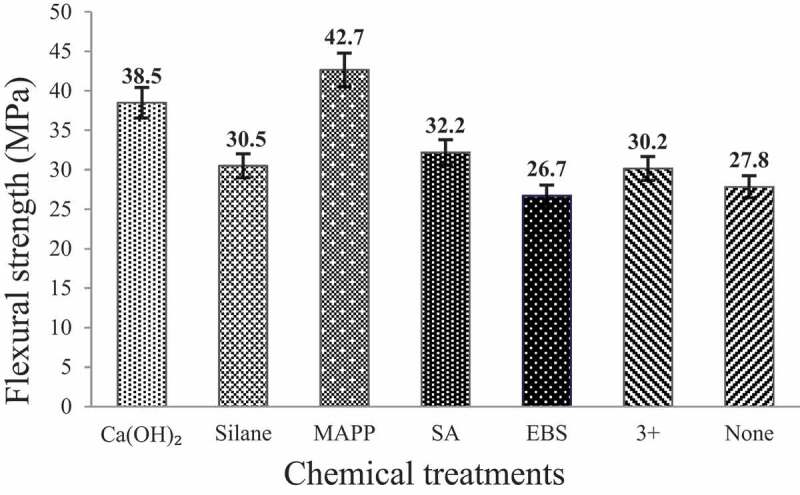
The effect of different chemical treatments on the flexural strength of CH reinforced composites.

The effect of various chemical treatment of CH fiber on the flexural strength of the reinforced composite is shown in Figure 9. The general data can be summarized as MA-g-PP > Ca(OH)_2_ > SA > silane > combination > untreated> EBS. It may be because MAPP makes the fiber structures between materials stronger and forms a network structure, which improves the surface stress of materials [45].

## 4. Conclusions

In this study, the control and processing of coffee hull fiber are examined from a feasibility, technical and theoretical basis for the first time. The results show that: incorporation of CH markedly improved the flexural and tensile properties of the reinforced HDPE matrix composites. Composites also demonstrated better resistance against water uptake as compared to other polymer matrix composites reinforced with other types of lignocellulosic fibers. At the same time indicated Ca(OH)_2_ and silane are used for treatment that best suits compatibilization process of the CH with the high density polyethylene in the reinforced composite. This material has great potential to efficient way for the committee to the use of waste.

## Highlights

- The feasibility of coffee hull reinforcement in thermoplastic composites is discussed;
- Improving the properties of Thermoplastic Composites by coffe hull;
- The influence of modifier on the composite was discussed and the best modification method was found;

It provides some useful data and methods for the utilization of lignocellulose.
